# Mass Spectrometry-Based Zebrafish Toxicometabolomics: A Review of Analytical and Data Quality Challenges

**DOI:** 10.3390/metabo11090635

**Published:** 2021-09-17

**Authors:** Katyeny Manuela da Silva, Elias Iturrospe, Chloe Bars, Dries Knapen, Steven Van Cruchten, Adrian Covaci, Alexander L. N. van Nuijs

**Affiliations:** 1 Toxicological Center, Department of Pharmaceutical Sciences, Faculty of Pharmaceutical, Biomedical and Veterinary Sciences, Campus Drie Eiken, University of Antwerp, Universiteitsplein 1, 2610 Antwerp, Belgium; elias.iturrospe@uantwerpen.be (E.I.); adrian.covaci@uantwerpen.be (A.C.); 2 Department of In Vitro Toxicology and Dermato-Cosmetology, Faculty of Medicine and Pharmacy, Campus Jette, Free University of Brussels, Laarbeeklaan 103, 1090 Brussels, Belgium; 3 Comparative Perinatal Development, Department of Veterinary Sciences, Faculty of Pharmaceutical, Biomedical and Veterinary Sciences, Campus Drie Eiken, University of Antwerp, Universiteitsplein 1, 2610 Antwerp, Belgium; chloe.bars@uantwerpen.be (C.B.); steven.vancruchten@uantwerpen.be (S.V.C.); 4 Zebrafishlab, Veterinary Physiology and Biochemistry, Department of Veterinary Sciences, Faculty of Pharmaceutical, Biomedical and Veterinary Sciences, Campus Drie Eiken, University of Antwerp, Universiteitsplein 1, 2610 Antwerp, Belgium; dries.knapen@uantwerpen.be

**Keywords:** zebrafish, metabolomics, mass spectrometry, analytical chemistry, quality assurance and quality control

## Abstract

Metabolomics has achieved great progress over the last 20 years, and it is currently considered a mature research field. As a result, the number of applications in toxicology, biomarker, and drug discovery has also increased. Toxicometabolomics has emerged as a powerful strategy to provide complementary information to study molecular-level toxic effects, which can be combined with a wide range of toxicological assessments and models. The zebrafish model has gained importance in recent decades as a bridging tool between in vitro assays and mammalian in vivo studies in the field of toxicology. Furthermore, as this vertebrate model is a low-cost system and features highly conserved metabolic pathways found in humans and mammalian models, it is a promising tool for toxicometabolomics. This short review aims to introduce zebrafish researchers interested in understanding the effects of chemical exposure using metabolomics to the challenges and possibilities of the field, with a special focus on toxicometabolomics-based mass spectrometry. The overall goal is to provide insights into analytical strategies to generate and identify high-quality metabolomic experiments focusing on quality management systems (QMS) and the importance of data reporting and sharing.

## 1. Introduction

Chemical safety is an emerging global concern due to the continuous growth of chemicals being produced and released into the environment. Therefore, toxicological assessments of short- and long-term exposure are crucial to evaluate the effects (*inter alia* latent and transgenerational) of a wide range of chemicals on human and environmental health [1]. As a result, in silico, in vitro and in vivo toxicological models are needed, as both short-term toxicity and transgenerational studies in humans are challenging, especially due to ethical constraints and critical confounding factors such as diet, different social exposures, and long lifespan (e.g., studies of toxic effects of low-dose and mixtures of chemicals) [2,3].

In this regard, the zebrafish (*Danio rerio*) (embryos, larvae, and adult fish) is a medium-to-high-throughput vertebrate toxicological model that is routinely used to provide relevant information regarding the acute and long-term effects of xenobiotics [4,5,6]. It encompasses multicellular biochemical processes and the conservation of several metabolic and physiological processes also found in mammals (e.g., mitochondrial metabolism, the role of lipid and glucose metabolism in embryonic development, endocrine axes regulating energy metabolism, physiology of the digestive system, etc.), illustrating the translational relevance of the model to study the effects of chemicals on metabolic pathways [4,7,8].

In the last 20 years, metabolomics, the study of small endogenous organic molecules (<1500 Da) in biological samples or organisms to find key metabolites in various biological processes, has been used as a promising alternative and/or additional tool to traditional toxicological assays [9,10]. Metabolic changes are dependent on several factors such as diet, sex, and disease [10]. Thus, a high degree of controlled conditions in toxicological models makes them suitable platforms for metabolomics with a wide range of applications, including chemical grouping, the discovery of points of departure from benchmarking dosing, and cross-species extrapolation of toxicity pathways [9,11]. For instance, metabolites and hence pathways affected by xenobiotic exposure can provide data to support the development of adverse outcome pathways (AOP). On the one hand, metabolite levels could provide relevant mechanistic information underlying key events at the molecular level [12]. However, in certain toxicological scenarios, specific metabolites could also be essential causally linked components of a particular toxicity pathway and thus be directly relevant as key events of the pathway. Read-across and grouping approaches based on structural similarity using metabolomics have been successfully applied to reduce the risk of uncertainty in the characterization of the toxicity profile of analog chemicals (e.g., 3-aminopropanol and 2-aminoethanol) [13].

The combination of the zebrafish model and metabolomics has shown promising results for toxicological and ecotoxicological applications (e.g., to study the effect of endocrine-disrupting chemicals) and for investigations on the mechanisms of metabolic diseases [4,9,14]. For instance, Ortiz-Villanueva et al. applied an untargeted metabolomics approach to investigate the effect of three endocrine disruptors (bisphenol A, perfluorooctane sulfonate and tributyltin) at sublethal doses on zebrafish embryos and found that similar biochemical pathways were affected by these three chemicals [15]. Similarly, perfluoroethercarboxylic acids (PFECA), a new generation of per- and polyfluoroalkyl substances (PFAS), were found to have similar metabolic profiles associated with the toxicity of discontinued PFAS in zebrafish embryos [16]. Further discussion of omics approaches for zebrafish as a screening model can be found in a recent review article by Lai et al. [5]. In this study, Lai et al. divided the experimental approaches for environmental toxicant studies into two major studies: transgenerational (epigenomics) and non-transgenerational studies (transcriptomics, proteomics, and metabolomics) showing how metabolomics experiments can generate complementary toxicity information that should be confirmed by functional studies and molecular biological experiments for validation.

The number of studies including metabolomics in zebrafish has sharply increased since 2011 (from four studies in 2011 to seventy in 2020 found in the PubMed database (https://pubmed.ncbi.nlm.nih.gov/, accessed on 13 July 2021), with many of them already indexed to the Zebrafish Information Network (ZFIN) (https://zfin.org/, accessed on 13 July 2021). Due to the complexity of data acquisition, processing, and interpretation, in addition to an appropriate study design, a quality management system (QMS) needs to be included and assessed during the entire metabolomics workflow. The current manuscript will address the analytical challenges and QMS of the zebrafish model (embryo, larvae, and adult fish) to demonstrate its application from a metabolomics perspective. Information was collected from recent literature indexed to PubMed and ZFIN search engines using combinations of the following keywords: metabolomics, lipidomics, zebrafish, and *Danio Rerio.* Recent articles (>2017) focusing on mass spectrometry toxicological applications using zebrafish were given preference. In addition, general metabolomics studies focusing on QMS and data analysis applications were included to introduce zebrafish researchers to the field and expand research on zebrafish toxicometabolomics.

## 2. Experimental Design 

### 2.1. Sample Collection

Different standardized fish toxicity test guidelines are available that could be useful for designing experiments allowing the collection of samples for metabolomics analyses. For example, Section 2 of the OECD’s (Organisation for Economic Co-operation and Development) Test Guidelines lists a number of different frequently used fish test guidelines (e.g., TGs 236, 210, 229, 234, and 203), often specifically tailored to the zebrafish as the model organism. Combined, these available test guidelines provide opportunities for studying both acute and chronic effects in all zebrafish life stages, ranging from embryos to larvae, juveniles, and adults, as well as transgenerational assays. For example, the Fish Embryo Acute Toxicity (FET) Test (TG 236) outlines an acute embryonic exposure design from 0–96 hpf, which covers the organogenesis period [17]. For zebrafish metabolomics studies, short-term exposure effects could be assessed in terms of hours (e.g., 2.5–4 h; as described in, e.g., TG 236 in embryos and TG 203 in adult fish) or medium- to long-term exposures in the larval period (e.g., 3–7 days) [15,18,19,20,21,22,23,24]. For chronic exposure, test guidelines are available for larval fish up to 30 days (e.g., TG 210) and for adult fish up to 20 or 60 days (e.g., TGs 229 and 234), when individually dissected fish tissues (e.g., liver, brain, intestinal samples, gonads) or blood samples could be analyzed [25,26,27].

#### 2.1.1. Euthanasia

A number of recent zebrafish metabolomics studies report ice as the anesthetic [21,28,29,30] and/or euthanasia [31] technique for adult zebrafish, larvae, and embryos. Euthanasia by rapid cooling is at present not allowed in the EU legislation, but, for example, in the United States, rapid chilling (2 to 4 °C, for 10–20 s) until loss of orientation and operculum movements followed by holding times in ice-chilled water is acceptable for euthanasia of zebrafish according to the Guidelines for the Euthanasia of Animals of the American Veterinary Medical Association (AVMA). While adults can be held for a minimum of 10 min in ice-chilled water, larvae of 4–7 days post-fertilization (dpf) should be kept for at least 20 min. However, rapid chilling alone has been shown not to be a reliable method for euthanasia of embryos < 3 dpf [32].

A second commonly used method is euthanasia with an overdose of tricaine methanesulfonate (MS-222) [33,34,35,36]. MS-222 is an anesthetic approved by the United States Food and Drug Administration (FDA) for temporary immobilization that is also used for euthanasia of fish by immersion in an MS-222 buffered solution (e.g., 200 mg/L, pH 7–7.5) [32,37]. The use of MS-222 to minimize suffering through suppression of the nervous system (inhibition of sodium channels) is accepted when the fish is kept for at least 5 min in the solution following the cessation of opercular movement and/or vestibulo-ocular reflex (EU Recommendation 2007/526/EC) [37]. However, similar to rapid cooling, MS-222 has been shown to be unreliable for euthanasia of early life stages [32]. Furthermore, MS-222 is rapidly metabolized and it can also cause changes in endogenous metabolites (e.g., increased glucose, catecholamines, and cortisol levels [37,38]). Therefore, more studies comparing MS-222 and rapid chilling methods for anesthesia and/or euthanasia are needed to understand their effects for metabolomics studies.

In addition to methodological considerations primarily intended for minimizing the potential effects of a selected euthanasia method on metabolomics profiles, ethical considerations have become important as well and should ideally be balanced against purely scientific arguments. While zebrafish embryos are not considered laboratory animals for ethical purposes in Europe (EU Directive 2010/63/EU; EU Directive 2012/707/EU) up to the free-feeding stage at 120 h post-fertilization (hpf), studies in adult fish require approval from an ethical committee according to international laws [39]. The method used for euthanasia must rapidly achieve unconsciousness and death with minimal pain and be performed by certified personnel capable of recognizing and confirming the death.

#### 2.1.2. Metabolism Quenching

Sample collection and preparation should be performed as quickly as possible to reduce the effect of additional metabolism and compound biotransformation [40]. Thus, quenching strategies are essential to ensure that the detected metabolites reflect the metabolism of the organism at the time of sampling. Importantly, for highly metabolically active matrices, such as tissues, any remaining enzymatic activity should be stopped by snap-freezing the sample with liquid nitrogen immediately after collection [40].

Although some metabolites, such as adenosine triphosphate (ATP) and glucose-6-phosphate, can turnover in terms of seconds, which is almost impossible to avoid, sodium metabisulfite and/or butylated hydroxytoluene can be added to reduce metabolite degradation [41]. It is worth mentioning that enzymatic and non-enzymatic lipid transformations (hydrolysis, oxidation, interspecies conversion) during sample collection, storage, and analytical steps are common processes that can produce misleading results (e.g., air oxidation of 1-palmitoyl-2-arachidonoyl-sn-glycero-3-phosphocholine (PAPC) [42], hydrolysis of glycerophospholipids by phospholipases A1 and A2 can increase levels of lysoglycerophospholipids and free fatty acid species [41], extraction with methanol/ethanol can lead to the formation of exogenous phosphatidylmethanol and phosphatidylethanol species mediated by phospholipase D [41,43]). As suggested by Ulmer et al. in a recent review on strategies and consideration for addressing lipid changes, lipid stability studies should be included during analytical method development [41]. The strategies mentioned in this latter work to avoid degradation or turn-over of metabolites include sample handling at low temperature (e.g., cold room), snap-freezing directly after collection, the use of additives to reduce enzymatic activity (e.g., 5 mM phenylmethanesulfonyl fluoride before sample extraction) and antioxidants (e.g., butylated hydroxyanisole, ascorbic acid, transferrin, deferoxamine) [41]. Preferably, freeze–thaw cycles should be limited, and analysis should be performed on fresh samples. However, analysis of fresh samples is often not feasible. Therefore, samples should be stored at −80 °C for as little time as possible and thawed only once for analysis [44]. 

### 2.2. Normalization and Variability

As a result of the low sample volume, embryos and larvae are usually pooled by treatment (e.g., control vs. exposed) for metabolomics analysis. Several studies reported the number of pooled individuals per sample group (e.g., 30–400) instead of weight [16,20,21,22,45,46]. Alternatively, Bai et al. performed metabolomics analysis in 168 hpf larvae using the weight of 25 mg per pooled sample group [47]. Dreier et al. normalized lipid concentrations in pooled larval zebrafish samples (*n* = 12) by the protein content in a targeted lipidomics study [34]. The authors mentioned that this normalization was an important step due to the low accuracy of weighing low amounts of sample. Untargeted metabolomics methods in zebrafish organs, e.g., pooled livers, also employed protein normalization to calculate the solvent volume to reconstitute the dried extracts before instrumental analysis [48]. However, there is not necessarily a strong linear correlation between protein content and metabolites, which can result in unreliable data [49]. The addition of extraction solvent depending on the tissue weight (>5 mg) can be a good strategy for pooled organs since they can still be measured with good accuracy using analytical balances [49,50,51]. Additionally, importantly, recent studies show that male and female zebrafish organs have different biochemical profiles. Pooling the samples using a 1:1 ratio based on sex can be challenging as females tend to be larger and could have an impact on the overall weight of pooled samples [52]. Moreover, adult female livers have a higher lipid content than males which is related to female reproductive function, and this should be taken into consideration while focusing on this specific organ [53]. Therefore, organ samples from males and females should preferably be processed as different groups for metabolomics. 

Complex organisms usually require more replicates than in vitro cell models to unveil meaningful biological information [44,54]. However, animals in controlled environments (e.g., standardized diet, day–night cycle times, temperature environment) require a smaller amount of replicates than epidemiological studies, but it is still advisable to keep the number of replicates to at least 6 per group [10,44]. For pooled embryos and larvae, a higher number of biological replicates should be considered to account for weight differences (e.g., Teng et al. used 8 replicates per group (one hundred embryos at 96 hpf) to evaluate the mechanisms of toxicity of a fungicide, flutolanil, on zebrafish development [24]). Nevertheless, pilot studies are crucial to estimate the variance originating from the analytical and pre-analytical workflows and to assess the need for a higher number of biological and/or technical replicates.

Trutschel et al. proposed an objective approach to determine variance at multiple levels of a metabolomics workflow with a pilot study using a hierarchical experimental design [55]. The authors used an experimental design with replicates at three levels (instrumental analysis; $\sigma_{instr}^{2}$, sample preparation; $\sigma_{prep}^{2}$ and biological $\sigma_{bio}^{2}$) to determine the minimum number of biological and technical (sample preparation and instrumental) replicates required to detect statistically significant features with a power hierarchical type of Student’s *t*-test. In addition, the authors also provided an R code to determine the achievable power if the number of biological and technical replicates is limited to a specific number based on pilot datasets.

### 2.3. Homogenization

Homogenization is a critical step for tissue samples [56]. Water and/or organic solvents are usually added to zebrafish samples and homogenization is usually performed using a bead beater with zirconium oxide beads (bead size 0.5 mm) for a few minutes (e.g., 4–6 min) [20,57,58]. For tissue samples, two beat cycles of 40 s at 6500 Hz followed by cooling in ice after each cycle can be sufficient to homogenize the samples [56]. For zebrafish samples, most studies do not describe in detail these latter parameters and their influence on the metabolite stability and sample homogenization. In addition to bead homogenization, the sample can also be kept in an ultrasound bath for 10–15 min [15,20]. This latter step should be carefully evaluated regarding the time, temperature, and frequency applied, since it can cause metabolite degradation [59].

### 2.4. Metabolite Extraction

Even in early development stages, zebrafish embryos and larvae contain a diverse and dynamic range of lipids (e.g., cholesterol, glycerophospholipids, triacylglycerols) and polar metabolites that are supplied by the yolk (e.g., amino acids and fatty acids) until approximately 120 hpf [60]. Extraction methods for untargeted metabolomics need to be tailored to capture the various chemical classes within the metabolome. However, there is no single extraction method that can cover the entire metabolome and lipidome. Some classes will be overlooked, while others will be enriched [61]. Depending on the goal of the study, the extraction can also be divided into “global” metabolomics [56], polar (primary) metabolomics [62], and lipidomics [63], which can be further subdivided and tailored for less abundant lipid classes, such as polyunsaturated fatty acids [64], steroids [65], or the epilipidome [66].

In addition, the extraction method is highly dependent on the sample matrix and the platform used for analysis. For instance, gas chromatography–mass spectrometry (GC-MS)-based metabolomics often require a derivatization step (e.g., oximation followed by silylation for a wide range of small polar metabolites [62] or single silylation for sterols [67]) after metabolite extraction to increase thermal stability and volatility. Sample preparation for zebrafish metabolomics using GC-MS has been recently reviewed by Yan et al. [68].

For liquid chromatography–mass spectrometry (LC-MS) analysis, liquid extraction (LE) is often used to extract and concentrate metabolites from zebrafish samples, as shown in Table 1. Single-phase extraction by adding more of the same solvent used for protein precipitation (e.g., methanol (MeOH) and/or acetonitrile (ACN) after removing the precipitate) is commonly applied [22,69]. However, to extract and dissolve non-polar lipids, a more apolar organic solvent, such as methyl tert-butyl ether (MTBE), dichloromethane (CH₂Cl₂), or chloroform (CHCl_3_), is usually required [34,70]. Single-phase methods are attractive because they reduce the time and complexity of the extraction, but they are also subject to a higher matrix effect and a smaller detection range due to the polarity diversity of the molecules in the metabolome (e.g., the LogP for citric acid is −1.64, while the predicted LogP (XLogP) for lysophosphatidylcholine (18:0) and triacylglycerol (48:0) are 6.6 and 22.1, respectively) [10,40,71,72].

In order to improve the efficiency of the extraction methods, two-phase LE or two-step LE can be used to increase the range of extracted molecules (e.g., LogP from −10 to up to 25). Popular two-phase extraction techniques for untargeted metabolomics include the classic Bligh and Dyer (CHCl_3_/MeOH/H_2_O, 2/2/1.8, *v/v/v*) and Matyash et al. (MTBE/MeOH/H_2_O, 10/3/2.5, *v/v/v*) extraction procedures [73,74]. The latter method replaces CHCl_3_ for the less toxic MTBE, which also causes a change in the location of the solvent layers (the organic layer is the upper phase for Matyash’s method) [10,75]. Two-phase extractions for a low amount of sample (e.g., 20 μL of plasma, 10^6^ cells, 2.5 mg of tissue) have been successfully applied using the above-mentioned (adapted) Bligh and Dyer, and Matyash techniques to analyze each phase with different LC-MS methods selected based on the LogP of the metabolites [54,76,77]. The successful application of these methods shows that reproducible extractions for metabolomics can also be obtained with low sample amounts. 

Sample preparation methods based on single-phase separation for zebrafish samples (Table 1) showed a clear tendency to favor polar metabolites detection, such as amino acids, tricarboxylic acid (TCA) cycle metabolites, with some of them also detecting polar lipids (e.g., acylcarnitines and some glycerophospholipids) [15,78], and interestingly, metabolites from the arachidonic acid metabolism, such as leukotriene B4 and prostaglandin E2 [78]. These latter compounds should be carefully evaluated with as much evidence as possible (reference standards, MS/MS spectra, retention time match, collision cross section values, isotopic pattern, mass defect) when detected by untargeted methods since they are usually present in low concentrations, and require specific analytical methods, including solid-phase extraction (SPE) extraction [35,79,80].

Strategies using a two-step solvent extraction showed promising results in terms of metabolite detection and data quality in untargeted methods for tissue samples [56]. Briefly, Want et al. extracted liver tissue samples using MeOH/H_2_O (1/1, *v/v*), followed by centrifugation and the supernatant removal. Subsequently, the solid precipitate was dissolved in CH_2_Cl_2_/MeOH (1/1, *v/v*) for the extraction of non-polar metabolites [56]. The application of two-step solvent extraction is an attractive approach for tissue samples, but it leads to an increased sample preparation time. The extraction requires two homogenization steps since the extract is centrifuged after the first extraction of the polar metabolites.

In addition to LE, SPE methods can be necessary to concentrate specific classes present in low concentrations, such as eicosanoids, oxylipins, and steroids [65,81]. Lebold et al., successfully applied an SPE method to fractionate three different lipid classes (i.e., sterols, fatty acids, and prenol lipids) in zebrafish embryos (24 hpf) using a modified polymer-based sorbent (strong anion exchange) [35]. Previously saponified samples with KOH were loaded onto the cartridges and the analytes were eluted with formic acid, ACN, and MeOH (5/47.5/47.5, *v/v/v*) based on hydrophobic (i.e., cholesterol), π–π bonding (i.e., α-tocopherol) and ionic interactions (i.e., polyunsaturated fatty acids (PUFAs)). SPE cartridges commonly used for lipid removal in fat-rich samples, such as Captiva-EMR (Agilent Technologies, Santa Clara, USA), can also be applied in a two-step method for extraction of polar and lipid metabolites. This latter cartridge traps lipids based on acyl chains, allowing small molecules to be eluted in a cleaner extract, which would allow higher concentration factors due to less interference of lipids. The second step includes the elution of lipids, in a different fraction, with a stronger organic solvent, such as CHCl_3_. However, its application for lipidomics and metabolomics workflow still needs to be evaluated, since most of the non-commercial applications are used for food analysis [82].

**Table 1:** Examples of recent studies using LC-MS metabolomics with zebrafish embryos, larvae, and tissues.

Collection Time	Sample per Replicate	Quenching/Storage	Extraction Solvent	Analysis	Reference
96 hpf *	30 pooled individuals	Snap-frozen with liquid nitrogen and stored at −80 °C	1 mL (MeOH/ACN/H_2_O, 40/40/20, *v/v/v*)	LC-HRMS Mostly polar metabolites, e.g., amino acids, and sugars	[20]
120 hpf	20 pooled individuals	Snap-frozen with dry ice and stored at −80 °C	1.7 mL (MeOH/H_2_O/CHCl_3_, 9/5/3, *v/v/v*)	LC-HRMS Mostly polar metabolites, e.g., amino acids and organic acids	[15]
168 hpf	50 mg (25 mg for metabolomics and 25 mg for lipidomics)	Not mentioned	Polar metabolites: 800 μL of (MeOH/ACN/H_2_O, 2/2/1, *v/v/v*) Lipids: 800 μL of −20 °C CH₂Cl₂/MeOH (3/1, *v/v*)	LC-HRMS Polar metabolites and lipids	[47]
120 hpf	15 pooled individuals	Snap-frozen and stored at −80 °C	590 μL (MeOH/ H_2_O/CHCl_3_, 15/15/29, *v/v/v*) + 10 uL of SPLASH LIPIDOMIX^®^)	2D-LC-HRMS Lipids	[70]
52 hpf *	10 pooled individuals	Snap-frozen with liquid nitrogen and stored at −80 °C	250 μL H_2_O for homogenization. Samples were freeze-dried and extracted with 80% MeOH (volume not specified).	LC-HRMS Mostly polar metabolites, e.g., purine metabolism and some lipids of the arachidonic acid metabolism	[78]
144 hpf	20 pooled individuals	10 μL of 13 mM sodium metabisulfite	450 μL of cold MeOH.	LC-MS/MS Mostly polar metabolites, e.g., kynurenine pathway metabolites, neurotransmitters	[18,19]
48 and 120 hpf	80 pooled individuals	Stored at −80 °C	Each 20 μL sample was extracted with 120 μL of cold 50% MeOH.	LC-HRMS Mostly polar metabolites, e.g., amino acids	[46]
120 hpf *	12 pooled individuals	Not mentioned	Samples were homogenized in 1 mL H_2_O + unknown amount of CH₂Cl₂	LC-MS/MS Lipids	[34]
24, 48, 72, and 120 hpf	15 pooled individuals	Stored at −80 °C	300 µL (MeOH/H_2_O, 80/20 *v/v*)	LC-HRMS Mostly polar metabolites, e.g., choline, betaine, methionine, glucose, and TCA cycle metabolites.	[57]
144 hpf	30 pooled individuals	Snap-frozen in liquid nitrogen	1 mL MeOH	LC-HRMS Polar metabolites, e.g., nucleosides, amino acids, and some lipid classes, e.g., sterol lipids, glycerophospholipids, sphingolipids	[22]
172 hpf *	200 pooled individuals (50 mg)	Snap-frozen in liquid nitrogen	400 µL (MeOH/H_2_O, 4/1, *v/v*)	LC-HRMS Polar metabolites, e.g., amino acids lipids, e.g., glycerophospholipids, arachidonic acid metabolism	[69]
24, 48, 72, and 120 hpf	10–15 pooled individuals	Stored at −80 °C	Saponification with alcoholic KOH with 1% ascorbic acid. The pH was adjusted to 2.5 with 12 mol/L HCl. Addition of 2.0 mL of hexane. Removed organic supernatant.	LC-HRMS(/MS) Docosahexaenoic acid, eicosapentaenoic acid, Arachidonic acid, and Linoleic acid	[57]
24 hpf	10 pooled individuals	Snap-frozen in liquid nitrogen and stored at −80 °C	SPE: Added samples to 2 mL 1% ascorbic acid in EtOH and 1 mL H_2_O. Saponification with 300 µL saturated KOH. Neutralization with 3 mol/L HCl to pH 7.5. Lipids were extracted/separated with Strata-X-A 33 mm Polymeric Strong Anion Exchange cartridges (200 mg/3 mL, Phenomenex) using different combinations of organic solvents: MeOH for Cholesterol, ACN for α-tocopherol, FA/ MeOH/ACN (5/47.5/47.5, *v/v/v*) for PUFAs.	LC-Single Quadrupole (MS) Free fatty acids Commercial Amplex Red Assay Kit (Life Technologies, Carlsbad, CA) Cholesterol LC-Electrochemical Detection α-tocopherol	[35]
24 and 36 hpf	200 and 100 pooled individuals	Snap-frozen in liquid nitrogen and stored at −80 °C	3 mL 66% MeOH	LC-HRMS(MS) Hydroxy-fatty acids, e.g., 7-HDHA, 10-HDHA, 14-HDHA, and 17-HDHA	[35]
72 and 168 hpf	15 pooled individuals	Stored at −80 °C	8 μL/mg cold MeOH and 3.2 μL/mg H_2_O. Added remaining solvents (8 μL/mg CHCl_3_ and 4 μL/mg H_2_O) to the homogenates. Final ratio: MeOH/H_2_O/CHCl_3_ (2/1.8/2, *v/v/v*). Dilution of upper layer 10-fold and transfer to 1.5 mL vial.	LC-MS/MS 22 amino acids + 22 polar metabolites (e.g., urea, betaine, uridine, inosine, xanthine)	[50]
Adult zebrafish **	Intestines (6 pooled individuals) 50 mg	Not mentioned	400 μL of MeOH/H_2_O (4/1, *v/v*)	LC-HRMS Polar metabolites and lipids, e.g., fatty acids, glycerophospholipids, carnitines	[29]
90 dpf	Liver (4 pooled individuals)	Snap-frozen and stored at −80 °C	Homogenized in approximately 1.2 mL of MeOH/H_2_O (4/1, *v/v*). Split into two fractions at a ratio of 5/1 (*v/v*) to analyze metabolites and lipids, respectively. Added MTBE, MeOH, and H_2_O to a final ratio of MTBE/MeOH/H_2_O (20/6/7, *v/v/v*) to the lipid fraction.	LC-HRMS Polar metabolites and lipids	[48]
Adult zebrafish **	Liver (8 pooled individuals)	Snap-frozen in liquid nitrogen and stored at −80 °C	Homogenized with 800 μL of MeOH and 200 μL of H_2_O. Collected 750 μL after centrifugation. Added another 200 μL H_2_O and 400 μL CHCl_3_.	LC-HRMS Mostly glycerophospholipids, amino acids, and fatty acids.	[83]

* Approximated value. ** Exact fish age during collection not specified.

### 2.5. Instrumental Analysis

The analysis of metabolites can be performed using untargeted, semi-targeted/quantitative, and/or targeted/quantitative approaches. In most cases, an untargeted method can be used as a first screening approach (e.g., hypothesis-generating), as this technique is less biased towards certain metabolite classes [10]. Untargeted methods can also be combined with semi-targeted approaches if there is prior knowledge of which metabolic pathways may be affected by a specific (exposure) condition. Once the metabolites of interest have been annotated, a targeted method (hypothesis-driven) can be used in order to detect changes in the concentration of specific metabolites using reference standards [10]. Metabolomics studies can be carried out using different analytical platforms including GC-MS, nuclear magnetic resonance (NMR), LC-MS, or a combination of them (e.g., ^1^H-NMR with LC-Orbitrap(MS)) [84]). An example of a multi-analytical platform used to study metabolite differences in zebrafish liver is the work of Ong et al. [52]. The authors used a combination of ^1^H-NMR for the analysis of monosaccharides, amino acids, and organic acids, GC-MS for cholesterol and fatty acids, and LC-MS for the analysis of lipids. However, more recent zebrafish toxicometabolomics studies (≥2017) reported using LC-HRMS (Orbitrap or QTOF) [15,28,29,31,33,36,48,51,85,86,87,88], followed by ^1^H-NMR [24,30,89,90,91,92,93] and GC-HRMS [21,94], while multi-analytical platform studies remain scarce.

In LC-MS analysis, the combination of hydrophilic interaction liquid chromatography (HILIC, e.g., bare silica, amide, diol, amido, zwitterionic columns) and reversed-phase liquid chromatography (RPLC, e.g., C18, C8, C30 columns) methods is one the most comprehensive strategies for untargeted metabolomics, providing a broad metabolite coverage [95,96,97]. Currently, C18 columns with sub-2-μm particle size are often used for untargeted metabolomics and lipidomics as a stand-alone technique, and rarely combined with HILIC [22,23,31,36,48,51,83,85,87,88,98]. Furthermore, the complementarity of HILIC to RPLC methods is a highly powerful strategy for polar metabolites that should not be overlooked, especially when a multiplatform strategy is not employed [97,99]. Depending on the stationary phase, HILIC columns can have different interaction mechanisms (e.g., hydrogen bonding, electrostatic interactions, hydrophilic partitioning) that can benefit the retention of polar metabolites, which would elute close to the void time in RPLC columns, and also allow the separation of lipids by the polarity of head groups [95,100]. Due to the recent development of robust columns and increasing knowledge on how to manipulate the retention of metabolites (e.g., solvent, salt modifiers, temperature, pH), amide and aminopropyl HILIC columns have also been used in zebrafish metabolomics studies as a stand-alone technique [15,20,28,86].

In addition, MS data can be acquired in positive and negative mode with electrospray ionization (ESI+ and ESI−, respectively) which benefits both acidic and basic functional groups [101]. Consequently, with the same chromatographic column, two datasets can be obtained (ESI+ and ESI-). If two chromatographic columns are used (e.g., HILIC and RPLC), four datasets are obtained which require parallel processing [97]. For instance, Hu et al. used a combination of RPLC (C18)-based ACQUITY UPLC HSS T3 (100 × 2.1 mm, 1.8 μm) and HILIC-based ACQUITY UPLC BEH Amide (100 × 2.1 mm × 1.7 μm) columns, both in positive ionization mode [87]. Keerthisinghe et al. used a HILIC Luna aminopropyl column (150 × 10 mm, 3 μm) for polar metabolites in positive and negative ionization modes and an RPLC (C18) Zorbax Eclipse Plus RRHD column (50 × 2.1 mm, 1.8 μm) in positive mode for lipidomics [48]. Recently, Xu et al. analyzed zebrafish embryos using different combinations of chromatographic methods to evaluate their metabolite coverage [70,102]. Their results highlighted the need for two methods, one HILIC based with an XBridge Amide column (150 × 2.1 mm × 3.5 μm) in ESI+ and one pentafluorophenyl Kinetex F5 column (150 × 2.1 mm × 2.6 μm) in ESI− to cover the polar metabolome (336 annotated metabolites) and one comprehensive two-dimensional (2D) liquid chromatography method with an EVO C18 column (100 × 2.1 mm × 2.6 μm) and a BEH HILIC column (50 × 2.1 mm × 1.7 μm) for comprehensive lipid profiling of zebrafish embryos (1784 annotated lipids). Nevertheless, 2D-LC analysis requires a long analysis time (approximately 170 min for this later study), which can consequently be considered not particularly suitable for large batches, solvent consumption, and sample stability.

Furthermore, the separation of isomers and isobars, and consequently, the acquisition of well-resolved fragmentation spectra is a challenge especially for lipidomics applications [103,104]. In order to obtain a less time-consuming but comprehensive platform, ion mobility spectrometry (IMS, a separation technique based on the mobility of ions through a buffer gas under the influence of an electric field) has been successfully integrated into LC-MS-based lipidomics and metabolomics workflows [96,105]. One of the key advantages of the IMS technique is that it can separate ions in milliseconds based on their shape and size, which is highly convenient for linking LC separations (minutes) and time-of-flight (TOF)-MS detection (microseconds) for the separation of E/Z isomers, *sn*-positional isomers, and increasing annotation confidence with the addition of collision cross section values [104,105].

### 2.6. Data Analysis

Data derived from metabolomics workflows are of great complexity since it usually englobes thousands of features (e.g., for LC-MS, an entity with an attributed *m/z*, retention time, fragmentation spectra, and a response signal). The workflow for untargeted data preprocessing includes several steps to obtain a feature signal response matrix (e.g., features in rows vs. samples in columns). These steps are dependent on the instrument (e.g., GC-MS, LC-MS, NMR) used to acquire the data. For MS-based instrumentation, if vendor-specific software is not used, data files need to be converted to an open file format (e.g., mzML, netCDF, ABF) to be further processed with open-source software packages, such as MS-DIAL, XCMS, MZmine, and OpenMS [106,107]. The software can be used to perform peak picking, deconvolution, alignment across samples, and in some cases, the same software can perform metabolite annotation with experimental and/or in silico libraries (e.g., MS-DIAL [108]). Peak picking, deconvolution, and alignment processes are performed to detect ions in a specific region of interest above pre-defined instrumental noise levels, to handle overlapping peaks, fragments and to align those signals across different samples.

Feature tables do not contain unique signals corresponding to a specific metabolite, but also redundant features (e.g., different isotopes, charges, and adducts in soft ionization techniques such as ESI and MALDI), background signals, etc. [109]. Computational techniques can address the challenge of isotope and adduct annotation, including the well-known R package CAMERA (based on peak grouping after retention time and peak shape correlation to form groups of ions, followed by annotation of possible isotopes and adducts) and the more recent web application MS-FLO (based on several parameters such as peak height, retention time alignment and mass similarities to detect adducts, isotopes and duplicate features in a preprocessed dataset) [109,110]. The number of software packages, databases for metabolite annotation, and processing tools increase every year, with the most recent developments compiled in a review paper by Misra [107].

Following data preprocessing, data cleaning (feature reducing) (e.g., remove background ions and features with low precision and detectability), signal drift correction, and imputation of missing values are commonly applied [111,112,113]. Then, data transformation and statistical analysis (univariate and/or multivariate techniques) are performed to identify relevant features for a specific condition, followed by further structure elucidation and biological interpretation. Depending on the type of statistical analysis (univariate/multivariate), different data pretreatment methods are necessary. Statistical analysis of metabolomics data is a complex workflow and requires tailored approaches. Recently, Blaise et al. published a comprehensive protocol for statistical analysis of metabolomics data, including scaling, normalization, outlier detection, statistical tests, power tests, and performance evaluation of models [114]. In addition, the authors made the scripts (Python 3 with Jupyter Notebooks), tutorials, and data freely available on GitHub (https://github.com/Gscorreia89/chemometrics-tutorials, accessed on 10 August 2021).

Importantly, independent of the data analysis workflow used, the Metabolomics Society Data Analysis Task Group proposed minimum requirements when reporting metabolomics data that can be used as a guideline for authors and reviewers. These requirements include preprocessing parameters (e.g., peak picking, deconvolution, alignment), pre-treatment strategies (e.g., normalization, scaling, transformation, missing value imputation, outlier detection), processing (e.g., model selected for analyzing data such as principal component analysis (PCA)), post-processing (e.g., back-transformation, visualization); validation (e.g., training, monitoring, and usage of a test set) [115]. However, they were proposed in 2007 and an update is required considering the advances in software and data sharing. For instance, interactive web-based computational laboratory notebooks (e.g., Jupyter Notebook) and cloud computing have emerged as a possible solution for data analysis transparency, open collaboration with the integration of codes, figures, tables, and user-friendly interfaces [116].

## 3. Quality Management System

As of 2021, metabolomics is considered a mature discipline with more than two decades of advancements in analytical workflows, software tools, and biological information in different biological systems (e.g., in vitro cells, plants, bacteria, animals) [10,44]. In parallel, guidelines to ensure data reliability and reproducibility have been proposed by the Metabolomics Standards Initiative (MSI) and the metabolomics Quality Assurance and Quality Control Consortium (mQACC) [117,118]. The MEtabolomics standaRds Initiative in Toxicology (MERIT) was created in 2017 to describe practical guidelines and minimal reporting standards for regulators to interpret the quality of metabolomics data in the context of regulatory toxicology [119]. Although the MERIT guidelines were not initially proposed for academic research, the definitions of commonly used terms in metabolomics (e.g., process blanks, system suitability QC, interlaboratory QC, level of confidence in metabolite annotation), scenarios for applications (e.g., metabolic points of departure (PODs), the discovery of chemical mode(s) of action and molecular key events (KEs), chemical grouping for read-across and cross-species extrapolation of toxicity pathways), data acquisition, and management strategies were described in a detailed and comprehensive manner and their use could be beneficial for toxicometabolomics research [119].

### 3.1. Quality Assurance (QA) and Quality Control (QC)

**Figure 1:**
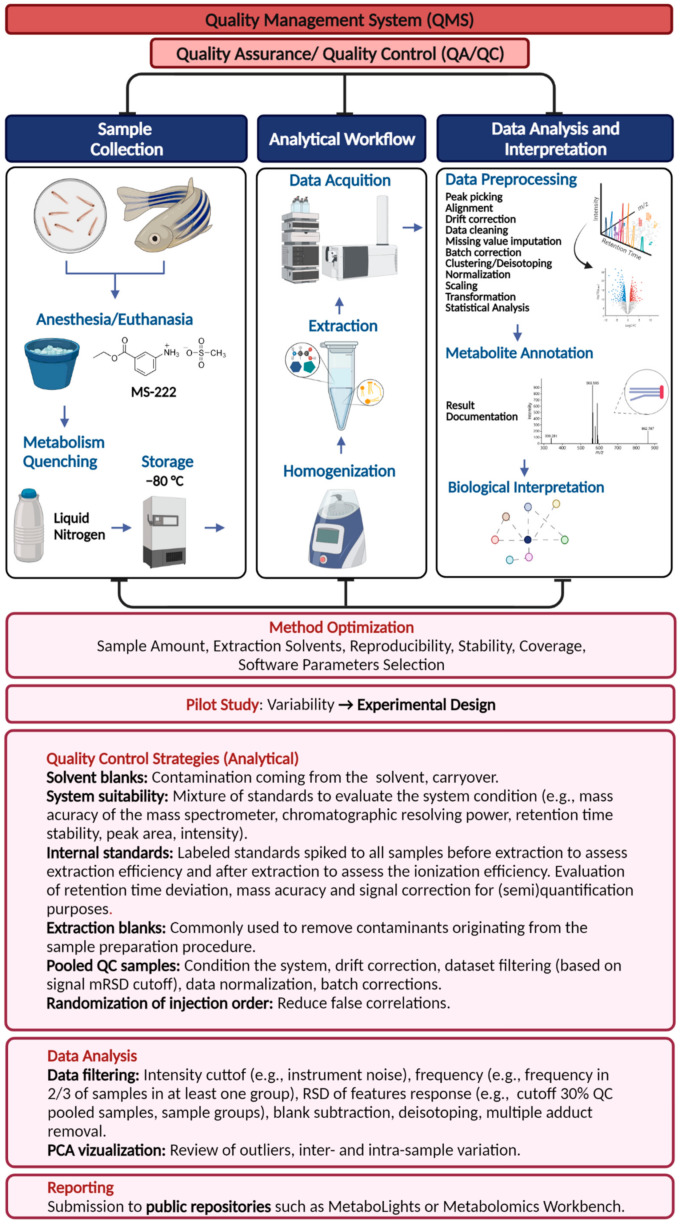
Quality management system strategies in the zebrafish metabolomics workflow.

Data quality assessment includes quality assurance (QA) and quality control (QC) measures to ensure data quality and reduce the risk of misinterpretation of results in a biological context (Figure 1). QA procedures include the development and continuous improvement of standard operating procedures for sample collection, preparation, data acquisition, and data processing. QC activities are undertaken during and after the experiment to monitor and report quality requirements. For metabolomics applications, these include the acquisition of blank extraction samples, intra-study QC pooled samples (e.g., mixed aliquots of biological samples representative of the entire sample set), spiking of samples with internal labeled standards to assess precision during the analysis, and reference materials for inter-laboratory and long-term studies [120]. However, there is currently no agreement on which quality metrics should be used and reported. 

The mQACC began to address the lack of guidelines and nomenclature by collecting detailed information on QA and QC practices used by different laboratories using LC-MS-based untargeted analysis [117]. Pooled QC samples were identified as one of the most commonly applied quality measurements in untargeted LC-MS-based metabolomics studies [117]. The importance of QC pooled samples for instrumental source conditioning, carry-over assessments, data filtering, signal correction, and determination of precision has been shown by recent software developments and applications [111,121,122].

Furthermore, the main consensus that resulted from the mQACC consortium group was the prospect of creating a set of minimum QA and QC practices for metabolomics. Meanwhile, the current guidelines for QA/QC management processes proposed by Broadhurst et al. can be used for zebrafish metabolomics studies, since they present good application and reporting practices, including the use of system suitability samples, process blanks (extraction blanks), pooled QC samples, QC conditioning samples, QC batch correction, and order of analytical batches [122]. Nevertheless, one of the most important aspects of QA/QC practices is the description of data acquisition and processing strategies, which includes feature-reducing strategies [115]. For instance, in the work of Broadhurst et al., the authors did not advise the use of pooled QC serial dilution to filter data based on correlations, since it does not consider a non-linear response, but they mentioned that further work is required to extend the use of this approach [122]. A recent application of the QC dilution series and advanced filtering proposed by Sands et al. showed that dilution QC series, besides its challenges (e.g., poor representation of low abundant metabolites, correct models for a response, account for matrix effects), can be used to estimate the linear range and analyte response and improve the data quality of global metabolic studies [123].

### 3.2. Level of Confidence in Metabolite Annotation

**Figure 2:**
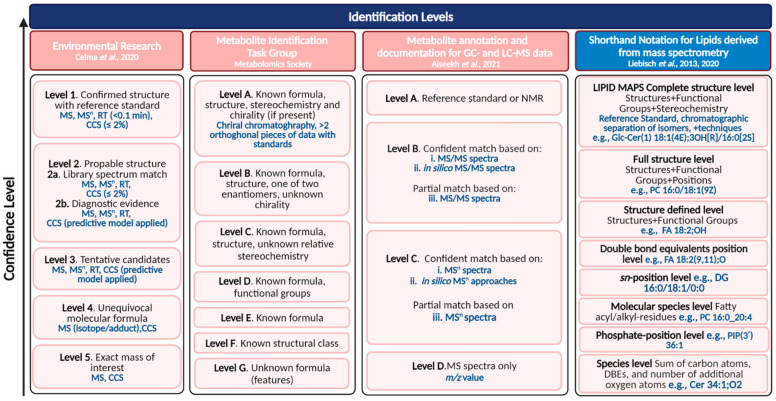
Overview of confidence levels in compound annotation using different scales. The figure was adapted from [125,126,127,128] and more detailed information about the mentioned confidence levels can be found in [124,125,126,127,128,129,131].

The importance of confidence in metabolite annotation for toxicometabolomics studies was highlighted by Malinowska et al. [124]. The authors proposed a framework for confidence levels required for metabolite annotation for different toxicological applications (e.g., application of metabolomics to derive points of departure require a higher confidence level in metabolite annotation than chemical grouping studies) using the earliest MSI confidence levels (1–4) definition [125]. More recently, in the context of mass spectrometry-based metabolomics, a simplified reporting workflow at the level of the processed data was proposed by Alseekh et al. [126] including guidelines for sample collection, extraction, storage, metabolite identification, and reporting (Figure 2). One of the main highlights of the latter work is the minimum ontology for metabolite documentation in research articles: analytical identifiers (retention time, theoretical monoisotopic mass, *m/z* of the adduct detected, *m/z* error (in ppm), MS/MS fragments, representative chromatograms, peak intensity, area for quantified data), and international identifiers for known metabolites (e.g., HMDB, KEGG, PubChem, LIPID MAPS). The authors proposed identification levels based on letters (A-D) with sublevels (i, ii, iii), as shown in Figure 2. However, these levels of confidence reflect more on the basic LC-MS information (e.g., retention time, MS/MS spectra) used to support the annotation rather than on the level of confidence based on the metabolite complete structure level.

The Metabolite Identification Task Group of the Metabolomics Society suggests confidence levels based on the metabolite complete structure level which in some cases requires additional techniques (e.g., ozonolysis or photochemical derivatization to determine the position of double bonds in glycerolipids). The MSI definition, within the metabolomics area, can be more robust towards instrumental advancements and the combination of different techniques to support annotation (e.g., the inclusion of collision cross section values derived from ion mobility spectrometry resulted in a new rank for confidence levels of identification of chemicals in environmental research [127]). 

The lipidomics community adopted a shorthand notation to standardize lipid nomenclature that supports the combination of different MS-derived techniques. This latter shorthand notation also reflects on the confidence levels [128]. For instance, *sn* positions can be determined by both IMS and chromatographic separation which results in the same confidence level for shorthand notation [129]. Often in MS-derived analysis, structure information is limited to level C (known formula and structure, unknown stereoisomers), hence it is important to document the information used to support annotation and use evidence-based nomenclature for data transparency [126,129,130].

### 3.3. Data Sharing

The submission of metabolomics studies to open access repositories, such as MetaboLights (https://www.ebi.ac.uk/metabolights/, accessed on 13 July 2021) and Metabolomics Workbench (https://www.metabolomics workbench.org, accessed on 13 July 2021) is considered as the gold standard for metabolomics reporting [126]. While submission requirements would benefit from standardization, data submitted to open repositories contribute to transparency and reproducibility [126,132]. Also, reanalysis of data can drive advances in computational analysis and the discovery of metabolites that were not found in the previous studies [133]. As of August 2021, there are 2418 metabolomics datasets publicly available in MetaboLights (849), Metabolomics Workbench (1543), and Metabolomic Repository Bordeaux (26) combined, which can be consulted via MetabolomeXchange (http://www.metabolomexchange.org/, accessed on 13 July 2021). However, only 6 datasets using the keywords “zebrafish” or “Danio rerio” were found (Table 2). This indicates that even though it is a highly recommended practice, articles applying metabolomics to zebrafish are not often submitted to these data repositories.

## 4. Conclusions and Perspectives

The specific debate of using omics data in the context of OECD test guidelines dates back for more than a decade and started with assessing the potential added value of transcriptomics data. From a toxicological perspective, metabolomics could provide a more detailed understanding of toxic mechanisms of chemicals at a molecular level. The value of metabolomics studies for different OECD fish test guidelines (e.g., TG 236, 210, 229, 234, and 203), which typically focus on the measurement of apical endpoints such as mortality, growth, and reproduction could become an important element of 21st century hazard and risk assessment strategies. Furthermore, dose-range finding experiments are necessary to determine relevant exposure scenarios given a specific experimental context and for a particular toxicological endpoint. Effect concentrations for metabolomics studies can differ from those observed at other levels of biological organization, meanwhile, it is the research question that determines the relevant exposure range. For example, the concentrations required to study metabolic changes associated with loss of reproductive capacity will be different from those changes associated with loss of equilibrium. Consequently, no general rules such as using the lowest observed effect concentration (LOEC) for a specific toxicological endpoint as maximum exposure level for zebrafish toxicometabolomics have been proposed in this work.

From an analytical chemistry perspective, fast quenching of samples using liquid N_2_ is necessary to avoid post-collection metabolic changes, while the use of antioxidants can help to preserve samples during preparation and storage at −80 °C. Extraction methods will depend on the class of metabolites under investigation since global metabolomics methods with full coverage of the metabolome even though desired, cannot be achieved. For comprehensive untargeted LC-MS-based metabolomics, liquid–liquid extraction to separate polar and non-polar metabolites followed by their analyses using different combinations of HILIC-HRMS and RPLC-HRMS has shown high potential. Depending on the laboratory infrastructure and available time, multi-analytical platform metabolomics can be used to improve metabolite coverage. Data acquisition parameters, data preprocessing, data pretreatment, statistical analysis, feature annotation, validation, and biological interpretation need to be carefully evaluated and it is recommended to report each step in detail according to the minimum requirements proposed by the Metabolomics Society Data Analysis Task Group. To ensure reliable and high-quality data, a quality management system is of vital importance. The current guidelines for QA/QC mentioned in this review with the usage of *inter alia* system suitability samples, process blanks, pooled QC samples, and QC conditioning samples, present good application, and reporting practices. In addition, confidence levels should be used for all annotated metabolites accompanied by the used confidence level system. Finally, submission of data to open repositories is necessary to improve transparency and reproducibility of obtained results and allow systematic comparisons of metabolites and pathways affected by specific classes of chemicals.

## Figures and Tables

**Table 2:** Zebrafish studies in publicly available repositories.

Title	Study	Sample	Analytical Technique	Example of Metabolite Classes Detected *	Repository	Reference
Metabolomics characterization of zebrafish larvae	Research article	Larvae	RPLC-MS and HILIC-MS	Hydroxy fatty acids, tricarboxylic acids, short-chain FA, folic acids, tetrahydrofolic acids	Metabolomics Workbench (ST001670)	[134]
Fasting wildtype, tfeb -/- knockout, and lmna -/- knockout metabolite profiling of adult zebrafish	Pilot study	Kidney, heart, muscle, and liver	RPLC-MS and HILIC-MS	-	Metabolomics Workbench (ST000584)	-
Zebrafish Metabolomics: Model for Environmental Metal Toxicity	Seed project	Larvae	NMR (^1^H, 700 MHz)	Acyl carnitines, Amino acids, Amino FA, Benzoic acids, Branched FA, Carboximidic acids, Cholines, Dialkylamines, Hydroxy FA, Imidazolines, Organic phosphoric acids, Primary alcohols, Saturated FA, Short-chain acids, Sulfones, TCA acids, Tertiary amines	Metabolomics Workbench (ST000365)	-
Plasticizers as obesogens in zebrafish	Feasibility study	Larvae	RPLC-MS	Amino acids, Amino FA, Xanthines, Butenolides, Benzoic acid esters, Catecholamines, Dicarboxylic acids, Dipeptides, Hypoxanthines, Monosaccharides, Phosphate esters, Pyrimidine deoxyribonucleosides, Pyrimidine ribonucleosides, Pyrimidines, Short-chain acids, Sugar alcohols, Sulfonic acids, TCA acids	Metabolomics Workbench (ST000556)	
Molecular structural diversity of mitochondrial cardiolipins	Research article	Whole body embryos and adults, head, tail	RPLC-MS	Cardiolipins (# of carbons 48–84)	MetaboLights (MTBLS636)	[135]
Lipidomics dataset of *Danio rerio* optic nerve regeneration model	Data in Brief	Adult optic nerve	RPLC-MS	Acyl carnitines, Ceramides, Dihydroceramides, Ceramide 1-phosphates, Phytoceramides, Sterol esters, Cardiolipins, Ubiquinones, Diradylglycerols, Fatty acids, Hexosylceramides, Glycerophosphocholines, Glycerophosphoethanolamines, Glycerophosphoglycerols, Glycerophosphoinositols, Glycerophosphoserines, Sphingomyelins, Triradylglycerols	Metabolomics Workbench (ST001725)	[136]

* The metabolite classes were obtained from RefMet [137].

## Data Availability

Data is contained within the article. The figures and tables of this manuscript are original. Graphical icons in Figure 1 and Figure 2 were provided by BioRender, license No. 2641-5211.
